# Information Filtering via Heterogeneous Diffusion in Online Bipartite Networks

**DOI:** 10.1371/journal.pone.0129459

**Published:** 2015-06-30

**Authors:** Fu-Guo Zhang, An Zeng

**Affiliations:** 1 School of Information Technology, Jiangxi University of Finance and Economics, Nanchang 330013, P.R. China; 2 Jiangxi Key Laboratory of Data and Knowledge Engineering, Jiangxi University of Finance and Economics, Nanchang, 330013, P. R. China; 3 School of Systems Science, Beijing Normal University, Beijing, 100875, P. R. China; Beijing University of Posts and Telecommunications, CHINA

## Abstract

The rapid expansion of Internet brings us overwhelming online information, which is impossible for an individual to go through all of it. Therefore, recommender systems were created to help people dig through this abundance of information. In networks composed by users and objects, recommender algorithms based on diffusion have been proven to be one of the best performing methods. Previous works considered the diffusion process from user to object, and from object to user to be equivalent. We show in this work that it is not the case and we improve the quality of the recommendation by taking into account the asymmetrical nature of this process. We apply this idea to modify the state-of-the-art recommendation methods. The simulation results show that the new methods can outperform these existing methods in both recommendation accuracy and diversity. Finally, this modification is checked to be able to improve the recommendation in a realistic case.

## Introduction

The recommender system is an important information filtering tool to exact the most relevant information for online users [1, 2]. Accordingly, it has been intensively investigated by researchers from computer science, physics and many other backgrounds [3–5]. Some of the algorithms have already been successfully applied to real online systems, such as *Amazon.com* and *Youtube.com*. With the recommender system, the page view and sale of the online products can be substantially increased [6]. Such improvement, however, depends a lot on the quality of the recommendation [7]. Therefore, the essential problem for the research on recommender system is how to develop an effective algorithm.

Even though there are various recommendation algorithms designed by computer scientists, such as collaborative filtering [8–10] and matrix factorization [11, 12], physicists take into account the personalization of the recommendation and design some diffusion-based algorithms which are able to achieve both high recommendation accuracy and diversity [5, 13]. One well-known method is the so-called hybrid method combining the mass diffusion and heat conduction processes on user-object bipartite networks [14]. The pure mass diffusion algorithm has a high recommendation accuracy while the heat conduction algorithm is outstanding in recommendation diversity, fusing these two algorithms thus gains high performance in both aspects [15]. Many extensions have been done to further enhance the performance of the diffusion-based recommendation algorithms [16–20]. Two representative ones are the preferential diffusion [21, 22] and biased heat conduction [23] algorithms.

Normally, these diffusion-based methods are based on two steps of diffusion on user-object bipartite networks. The diffusion starts by assigning one unit of resource on each object selected by the target user who we want to do recommendation to. In the first step, the resource diffuses to the users who selected the same objects as the target user. In the second step, the resource diffuses to these users’ selected objects. The objects with the highest final resource will be recommended to the target user. In the diffusion-based methods, both steps are based on the same diffusion rule. In the literature, it has already been shown that the structural properties of nodes of distinct types in bipartite networks can be completely different [24]. A recent paper has already combined the diffusion starting from the user side and the object side to solve the cold start problem in link prediction and spurious link detection [25]. Inspired by these works, in this paper we propose to design the rule of these two diffusion steps differently to improve the recommendation efficiency.

We first empirically analyze some online bipartite networks. We find that there is indeed significant difference in structural properties between the two types of nodes in these networks. Based on the well-known hybrid recommendation method [15], we propose a heterogeneous diffusion method in which each diffusion step is controlled by a separate parameter. Our results show that the new method can outperform the hybrid method in both recommendation accuracy and diversity. The idea of the heterogeneous diffusion is further extended to the preferential diffusion [21, 22] and biased heat conduction [23] algorithms, and similar improvement is observed. As the heterogeneous diffusion method requires to introduce an additional parameter, it may cause the problem of over-fitting [26], i.e. the method has too many parameters that only capture noise instead of the underlying relationship. In order to avoid the problem of over-fitting, we finally verify the heterogeneous diffusion method in the three-fold data division with a learning process [27].

## Data

To test the performance of the recommendation methods, we use three benchmark data sets. The Movielens data set [28] consists of 1682 movies (items) and 943 users who can vote for movies with five level ratings from 1 (i.e., worst) to 5 (i.e., best). According to the literature [21, 23], we only consider the ratings higher than 2. After coarse gaining, the data contains 82520 user-item pairs. The Netflix data set [29] is a random sampling of the whole records of user activities in *Netflix.com*. It consists of 10000 users, 6000 movies, and 824802 links. Similar to MovieLens, only the links with ratings of 3 and above are considered [15]. After data filtering, there are 701947 links left. The third data set is called RYM which was obtained by downloading publicly-available data from the music ratings website *RateYourMusic.com*. The data has 33786 users and 5381 objects with 613387 links. The data itself is unary, i.e. a user has either collected a web link or not. The data used in this paper can be found in S2 file.

## Empirical Analysis

**Fig 1:**
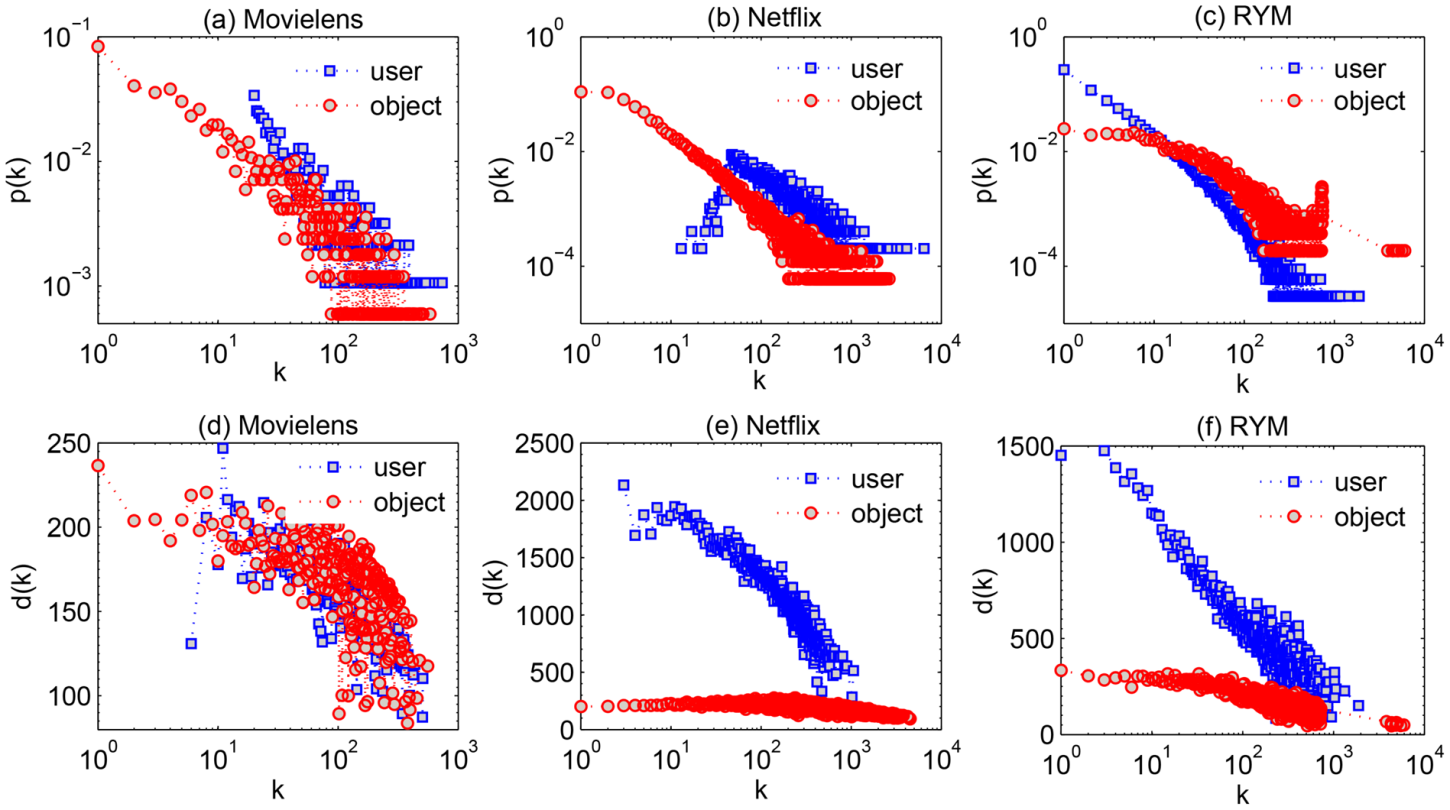
The degree distribution of users and objects in (a) Movielens, (b) Netflix and (c) RYM networks. (d), (e) and (f) are *d*(*k*) vs *k* in Movielens, Netflix and RYM networks, respectively. For the blue curve, *k* denotes the degree of users and *d*(*k*) denotes the average degree of the neighboring objects of these users. For the red curve, *k* denotes the degree of objects and *d*(*k*) denotes the average degree of the neighboring users of these objects.

We first consider the degree distribution. In Fig 1(a)(b)(c), one can see that the degree distribution of both types of nodes have broad degree distribution. However, the broadness of the degree distribution is significantly different between user nodes and object nodes, especially in the Movielens and RYM data sets. The second property we considered is the degree correlation between neighboring nodes. Instead of using the assortativity coefficient [30], we here calculate the neighbor connectivity to capture this property [31]. We denote *k*_*i*_ as the degree of user *i* and *d*_*i*_ as the average degree of the objects selected by user *i*. All the users with the same degree *k* are selected and their *d* values are averaged to obtain *d*(*k*). In Fig 1 (d)(e)(f), we present *d*(*k*) as a function of *k* in different data sets. Clearly, *d*(*k*) is decreasing with *k*, which indicates that high degree users tend to select unpopular objects while small degree users tend to select popular objects (see S1 file for more detailed explanation). Similarly, we can plot *d*(*k*) versus *k* from the object side. A decreasing function is also observed in this case. However, the slopes of the user-based curve and object-based curve differ from each other. Specifically, the negative correlation between *d*(*k*) and *k* is less obvious in the object-based curve. The above results evidently show that the structural properties of user nodes and object nodes are different in these online bipartite networks.

## Method

An online commercial system can be modeled by a bipartite network, where users and objects are characterized by two distinct kinds of nodes. The bipartite network is characterized by an adjacency matrix *A* where the element *a*_*iα*_ equals 1 if user *i* has collected object *α*, and 0 otherwise. The number of users and items is denoted as *N* and *M*, respectively. Consistent with the literature, we use Latin and Greek letters, respectively, for user- and object-related indices.

As mentioned above, we will take into account three recommendation algorithms: the hybrid method [15], the preferential diffusion method [21], and the biased heat conduction [23]. The hybrid method is a combination of the mass diffusion [32] and heat conduction [14] algorithms with a tunable mixing parameter λ. We first introduce the heterogeneous hybrid diffusion method (short for H-Hybrid). The basic idea is that the hybrid parameter λ should be different in two diffusion steps. In particular, for the target user *i* who we will recommend objects to, each of *i*’s collected object is assigned with one unit of resource. The resource of each object then distributes to all the neighboring users who have selected this object. User *j* receives the sum over all *i*’s collected objects:

$$\begin{array}{c} f_{ij}=\sum_{\alpha =1}^{M}\frac{a_{i\alpha }a_{j\alpha }}{k_{\alpha }^{\lambda_{1}}k_{j}^{1-\lambda_{1}}}, \end{array}$$ (1)

where *k*_*α*_ is the degree of object *α* and *k*_*j*_ is the degree of user *j*. In the second step of diffusion, each user distributes their resource back to the object side. The final resource of object *β* is

$$\begin{array}{c} f_{i\beta }=\sum_{j=1}^{N}\frac{a_{j\beta }f_{ij}}{k_{j}^{\lambda_{2}}k_{\beta }^{1-\lambda_{2}}}. \end{array}$$ (2)

**Fig 2:**
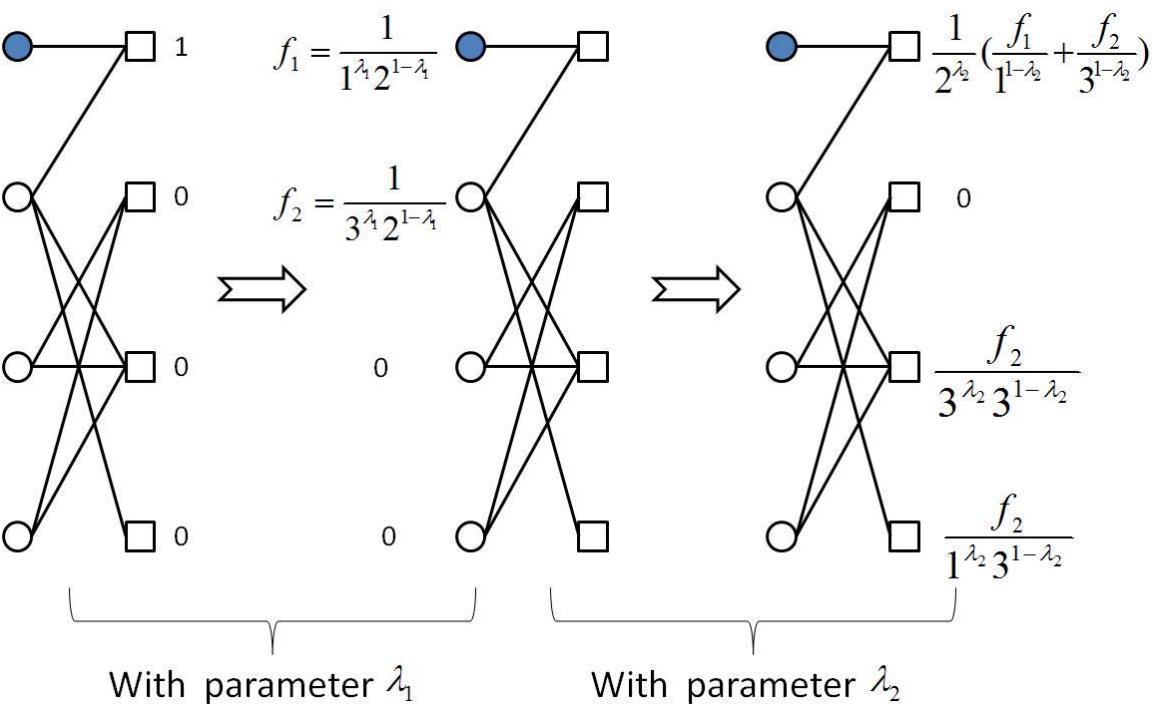
The illustration of the H-Hybrid method. Users and items are marked with circles and squares, respectively. Shaded circles indicate the target user for whom recommendation is done.

The parameter λ_1_ and λ_2_ adjust the relative weight between the heat conduction algorithm to the mass diffusion algorithm. With them increasing from 0 to 1, the algorithm changes gradually from the heat conduction algorithm to the mass diffusion algorithm. The H-Hybird method is illustrated in Fig 2. When λ_1_ = λ_2_ the method reduces to the original hybrid method (short for O-Hybrid). The recommendation list for the target user *i* is obtained by sorting all items according to *f*_*iβ*_ in a descending order.

A similar idea can be applied to the preferential diffusion and biased heat conduction methods. The original preferential diffusion (denoted as O-PD) [21] is also based on two steps of diffusion process on user-object bipartite networks. Again, for the target user *i*, each of *i*’s collected object is assigned with one unit of resource. In the first step of the heterogeneous preferential diffusion (H-PD) method, the resource is diffused to the users with

$$\begin{array}{c} f_{ij}=\sum_{\alpha =1}^{M}\frac{a_{i\alpha }a_{j\alpha }}{{ℳ}_{1}k_{j}^{-\epsilon_{1}}}, \end{array}$$ (3)

where ${ℳ}_{1}=\sum_{l=1}^{N}a_{l\alpha }k_{l}^{-\epsilon_{1}}$. In the second step, the resource is diffused back to the objects with

$$\begin{array}{c} f_{i\beta }=\sum_{j=1}^{N}\frac{a_{j\beta }f_{ij}}{{ℳ}_{2}k_{\beta }^{-\epsilon_{2}}} \end{array}$$ (4)

where ${ℳ}_{2}=\sum_{\gamma =1}^{M}a_{j\gamma }k_{\gamma }^{-\epsilon_{2}}$ is a normalization factor. Note that when *ϵ*_1_ = *ϵ*_2_, the H-PD method reduces to the O-PD method.

The original biased heat conduction (O-BHC) is very similar to the preferential diffusion method. In the heterogeneous biased heat conduction (H-BHC) method, the equation for the first step is

$$\begin{array}{c} f_{ij}=\sum_{\alpha =1}^{M}\frac{a_{i\alpha }a_{j\alpha }}{k_{j}k_{\alpha }^{-\gamma_{1}}}, \end{array}$$ (5)

and in the second step, the resource diffuses to the objects in a biased way as

$$\begin{array}{c} f_{i\beta }=\sum_{j=1}^{N}\frac{a_{j\beta }f_{ij}}{k_{\alpha }k_{j}^{-\gamma_{2}}} \end{array}$$ (6)

Again, when *γ*_1_ = *γ*_2_, H-BHC degenerates to O-BHC.

## Metrics

To test the performance of above methods, the real network data is randomly divided into two parts: the training set *E* ^*T*^ contains 90% of the links and the remaining 10% of links constitutes the probe set *E* ^*P*^. The recommendation algorithms run on *E* ^*T*^, while *E* ^*P*^ is used to evaluate the recommendation results.

An effective recommendation should be able to accurately find the items that users like. In order to measure the recommendation accuracy, we make use of *ranking score* (*RS*). Specifically, *RS* measures whether the ordering of the items in the recommendation list matches the users’ real preference. As discussed above, the recommender system will provide each user with a ranking list which contains all his uncollected items. For a target user *i*, we calculate the position for each of his links in the probe set. If one of his uncollected item *α* is ranked at the 5th place and the total number of his uncollected items is 100, the ranking score *RS*_*iα*_ will be 0.05. In a good recommendation, the items in the probe set should be ranked higher, so that *RS* will be smaller. Therefore, the mean value of the *R* over all the user-item relations in the probe set can be used to evaluate the recommendation accuracy as

$$\begin{array}{c} RS=\frac{1}{|E^{P}|}\sum_{i\alpha \in E^{P}}RS_{i\alpha }. \end{array}$$ (7)

The smaller the value of *RS*, the higher the recommendation accuracy.

In reality, online systems only present the top part of the recommendation list to users. Therefore, we consider another more practical recommendation accuracy measurement called *precision*, which only takes into account each user’s top-*L* items in the recommendation list. For each user *i*, the precision of recommendation is calculated as

$$\begin{array}{c} P_{i}\left(L\right)=\frac{d_{i}\left(L\right)}{L}, \end{array}$$ (8)

where *d*_*i*_(*L*) represents the number of user *i*’s deleted links contained in the top-*L* places in the recommendation list. For the whole system, the precision *P*(*L*) can be obtained by averaging the individual precisions over all users with at least one link in the probe set. The higher the value of *P*(*L*), the better the recommendations.

Predicting what a user likes from the list of the most popular objects is generally easy in recommendation, while uncovering users’ very personalized preference (i.e. uncovering the unpopular items in the probe set) is much more difficult and important. Therefore, diversity should be considered as another significant aspects for recommender systems besides accuracy. In this paper, we employ two kinds of diversity measurement: *personalization* and *novelty*.

The personalization mainly considers how users’ recommendation lists are different from each other. Here, we measure it by the Hamming distance. We denote *C*_*ij*_(*L*) as the number of common items in the top-*L* place of the recommendation list of user *i* and *j*, their hamming distance can be calculated as

$$\begin{array}{c} D_{ij}\left(L\right)=1-\frac{C_{ij}\left(L\right)}{L}. \end{array}$$ (9)

*D*_*ij*_(*L*) is between 0 and 1, which are respectively corresponding to the cases where *i* and *j* have the same or an entirely different recommendation list. By averaging *D*_*ij*_(*L*) over all pairs of users, we obtain the mean hamming distance *D*(*L*). The more the recommendation list differs from each other, the higher the *D*(*L*) is.

The novelty measures the average degree of the items in the recommendation list. For those popular items, users may already get them from other channels. However, it is hard for the users to find the relevant but unpopular item. Therefore, a good recommender system should prefer to recommend small degree items. The metric *novelty* can be expressed as

$$\begin{array}{c} I_{i}\left(L\right)=\frac{1}{L}\sum_{\alpha \in O^{i}}k_{\alpha } \end{array}$$ (10)

where *O* ^*i*^ represents the recommendation list for user *i*. A low mean popularity *I*(*L*) for the whole system indicates a high novel and unexpected recommendation of items.

## Results

**Fig 3:**
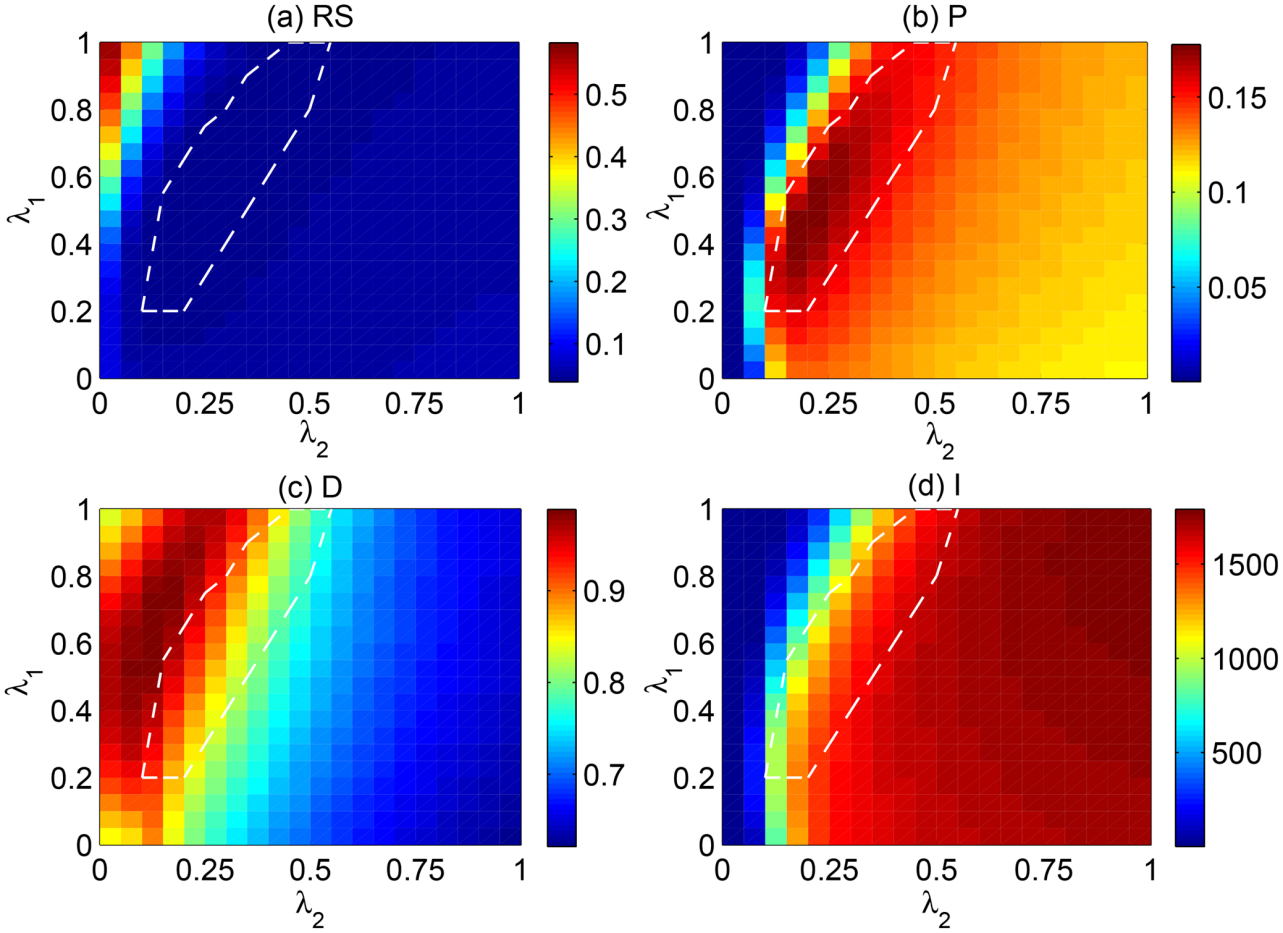
The (a) Ranking score, (b) Precision, (c) personlization and (d) novelty of the H-Hybrid method in parameter space (λ_1_, λ_2_) in Netflix network. The dashed line marks the region where RS is better than the RS value achievable with O-Hybrid method.

We first investigate the performance of the H-Hybrid method in the parameter space (λ_1_, λ_2_). The results on Netflix data set are shown in Fig 3. We checked that the results are consistent in Movielens and RYM data sets. Fig 3(a)(b) show the results of ranking score and precision of the H-Hybrid method. One can see from the heat maps that a minimum *RS* and a maximum *P* can be achieved. The optimal parameters for the minimum *RS* and maximum *P* are approximately the same, i.e. around $\lambda_{1}^{*}=0.45$ and $\lambda_{2}^{*}=0.25$. An interesting observation here is $\lambda_{1}^{*}\neq \lambda_{2}^{*}$, which confirms that the optimal recommendation accuracy is achieved when the parameters for the two diffusion steps are different. Fig 3(c)(d) present the results of personalization and novelty of H-Hybrid. It is clear that λ_2_ dominates the performance of the H-Hybrid method on recommendation diversity. However, the effect of λ_1_ shouldn’t be completely neglected. In fact, when λ_1_ is close to 1, the parameter range in which λ_2_ achieves high recommendation diversity becomes larger. There is no optimal parameters for both accuracy and diversity. As the accuracy is in general more important than diversity in recommendation, the optimal parameters in this paper are determined when the optimal recommendation accuracy *RS* is achieved.

**Fig 4:**
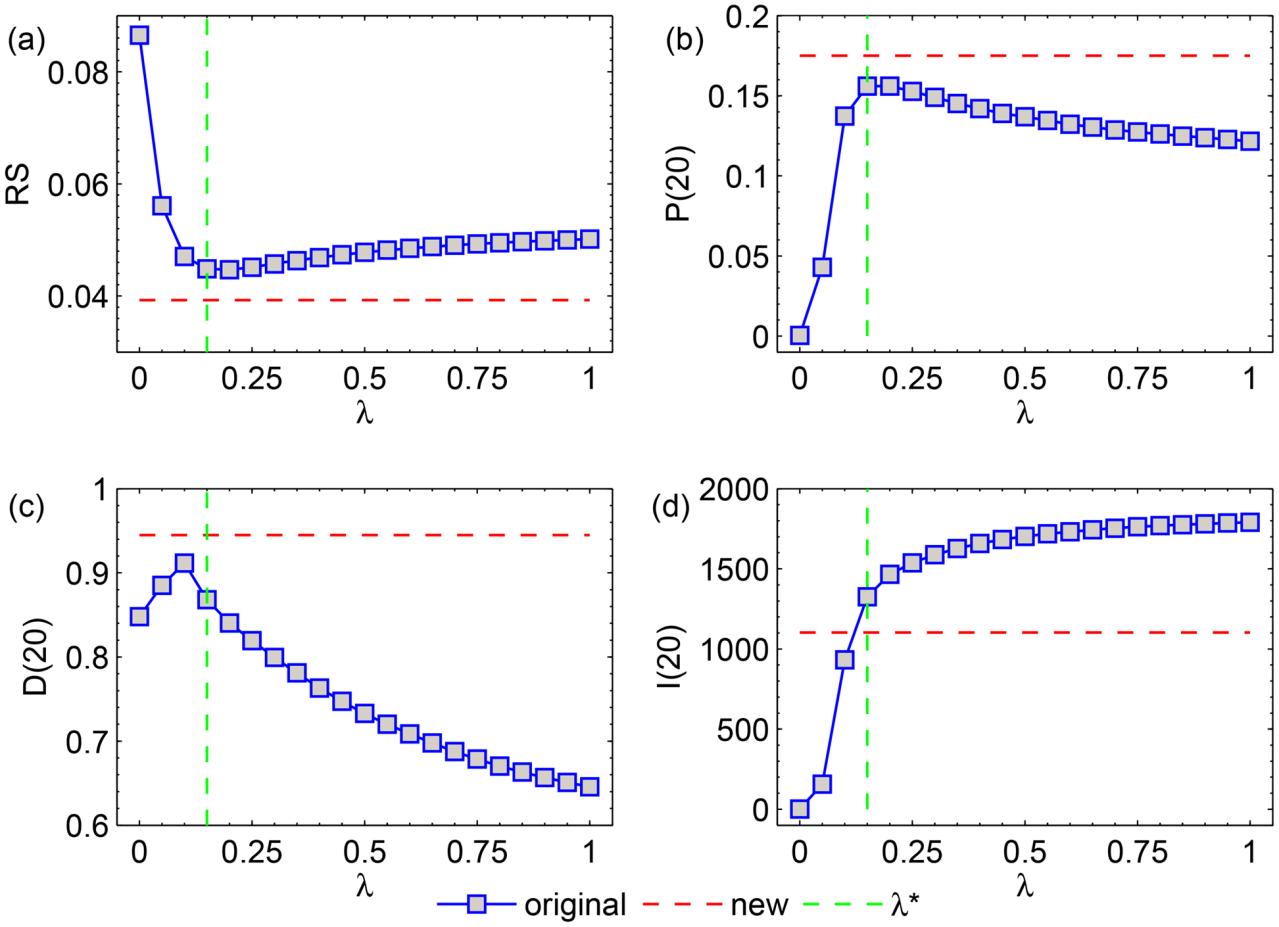
The (a) Ranking score, (b) Precision, (c) personlization and (d) novelty of the O-Hybrid method as a function of λ in Netflix network. The green lines mark the optimal λ* of the O-Hybrid method and the red lines mark the optimal results of the H-Hybrid method.

In order to show in detail the advantage of H-Hybrid over O-Hybrid, we present in Fig 4 some curves from the heat maps in Fig 3. The blue curves are the results of the recommendation metrics versus the parameter λ in the O-Hybrid method, which are basically the diagonals in the heat map in Fig 3. Consistent with Ref. [15], we observe an optimal recommendation accuracy (in both *RS* and *P*) when λ is tuned. The green dashed lines mark the optimal λ when the optimal recommendation accuracy (*RS*) is achieved in O-Hybrid. Moreover, we mark the optimal *RS* and *P* of the H-Hybrid method by the red dashed lines ($\lambda_{1}^{*}=0.45$ and $\lambda_{2}^{*}=0.25$). One can see that the H-Hybrid method can substantially outperform the O-Hybrid method in both *RS* and *P*. More specifically, the RS* in the O-Hybrid method is 0.0447 while the RS* in the H-Hybrid method can be as small as 0.0395. The improvement is 11.63%. For precision, P* is 0.1561 in O-Hybrid and P* is 0.1775 in H-Hybrid. The improvement of *P* is 13.71%. Fig 4(c)(d) show the results of recommendation diversity. Clearly, H-Hybrid recommendation is much more personalized and novel than O-Hybrid, with 9.0% improvement in *D* and 12.35% improvement in *I*.

In order to study the method on sparser data set, we consider the case where the real data is divided into probe set with 50% links and training set with 50% links. The results show that our method can still outperform the traditional recommendation method even under the sparse data. However, the advantage of our method becomes smaller when the training set becomes further sparser. This is natural because when the available information is limited, the recommender system cannot extract enough information of users’ preference. Therefore, the recommendation accuracy of even very outstanding recommendation algorithm cannot be good. Besides the O-Hybrid, O-PD, O-BHC methods, we also compare our methods with a more recent method called Directed Weighted Conduction (DWC) method (See table A in S1 file). We find that DWC can indeed outperform the heterogeneous diffusion (H-Hybrid, H-PD, H-BHC) in diversity, but some amount of recommendation accuracy is sacrificed. Finally, the computational complex of the diffusion-based algorithms (H-Hybrid, H-PD, H-BHC) is $O(N{\bar{k}}_{u}{\bar{k}}_{o})$ where ${\bar{k}}_{u}$ and ${\bar{k}}_{o}$ are the mean degree of users and items, respectively. It is actually much smaller than that of the widely-used item-based collaborative filtering (e.g. its computational complexity is *O*(*N* ^2^ *M*)). Therefore, we believe that the methods in this paper can also be applied to large network and meaningful in practical use.

**Fig 5:**
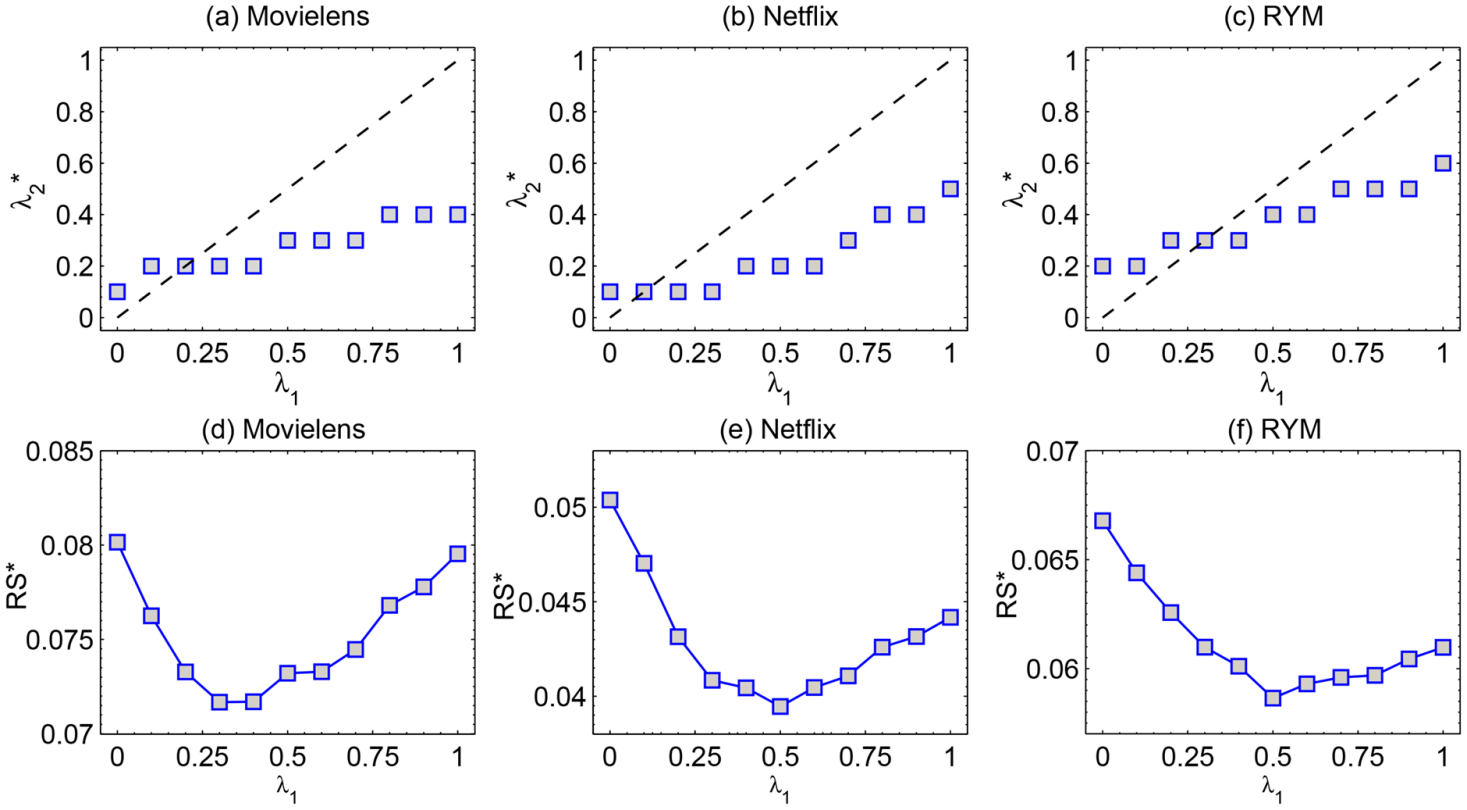
$\lambda_{2}^{*}$ vs λ_1_ in (a) Movielens, (b) Netflix and (c) RYM data. The line corresponding to λ_1_ = λ_2_ is plotted to guide eyes. In (d)(e)(f), the minimum RS* is obtained for $\lambda_{2}^{*}$ of the upper panels.

We further study the relation between $\lambda_{1}^{*}$ and $\lambda_{2}^{*}$. We tune λ_1_ from 0 to 1. For each λ_1_, we calculate the optimal $\lambda_{2}^{*}$ that results in an minimum *RS*. Accordingly, we show $\lambda_{2}^{*}$ vs λ_1_ in Fig 5(a)(b)(c). The dashed line in these figures is λ_1_ = λ_2_. Generally, $\lambda_{2}^{*}$ increases with λ_1_, but the curve doesn’t overlap with λ_1_ = λ_2_. When λ_1_ is small, $\lambda_{2}^{*}>\lambda_{1}$, and vice versa. The results can be understood easily. As shown in ref. [33], heat conduction process (i.e. λ = 0) tends to give high score to small degree nodes while mass diffusion algorithm (i.e. λ = 1) is in favor of high degree nodes. If the H-Hybrid method is assigned with a small λ_1_, the first step will be mainly based on heat conduction algorithm and small degree users will obtain high resource. A large λ_2_ means the second step is dominated by the mass diffusion algorithm and the popular objects selected by these small degree users should be recommended to the target user. On the other hand, if a large λ_1_ is used, a relatively smaller λ_2_ is needed.

In further support of the advantage of the H-hybrid method, we present the minimum RS* obtained by tuning λ_2_ when λ_1_ is given in Fig 5. Each λ_1_ is corresponding to a RS*. The dependence of RS* on λ_1_ is reported in Fig 5(d)(e)(f). One can see that RS* can be further reduced by tuning λ_1_, indicating the importance of λ_1_ in the H-Hybrid method. The above analysis is mainly based on the H-Hybrid method in Netflix data. More detailed values of other data sets and other methods are presented in Table 1. It is clear that all the diffusion-based recommendation algorithms can be improved by the idea of heterogenous diffusion. In Movielens and RYM, the best algorithm is the HPD. In Netflix, the best algorithm is H-Hybrid. These results also highlight the fact that there is no universally good algorithm, the best algorithm can vary from one system to another. It is therefore a crucial task to identify the most suitable algorithm for each online system when it comes to real applications.

**Table 1:** The results of all the metrics for different recommendation algorithms. The entries corresponding to the best performance over all methods are emphasized in black.

Network	Method	*RS*	*P*(20)	*H*(20)	*I*(20)
Movielens	O-Hybrid	0.0733	0.1545	0.8735	230.9
	H-Hybrid	0.0717	0.1573	0.8919	221.4
	PD	0.0703	0.1602	0.8831	225.8
	H-PD	**0.0701**	**0.1621**	**0.8905**	**222.5**
	BHC	0.0753	0.1510	0.8603	238.4
	H-BHC	0.0741	0.1544	0.8833	230.3
Netflix	O-Hybrid	0.0447	0.1561	0.8404	1466
	H-Hybrid	**0.0395**	**0.1775**	**0.9160**	1285
	O-PD	0.0406	0.1485	0.8486	1324
	H-PD	0.0405	0.1461	0.9088	**1024**
	O-BHC	0.0474	0.1522	0.8550	1365
	H-BHC	0.0448	0.1708	0.9402	1085
RYM	O-Hybrid	0.0606	0.0727	0.9239	1060
	H-Hybrid	**0.0586**	0.0750	0.9326	1012
	O-PD	0.0588	0.0755	0.9359	987.3
	H-PD	0.0588	**0.0755**	**0.9359**	987.3
	O-BHC	0.0651	0.0645	0.9281	966.7
	H-BHC	0.0646	0.0665	0.9342	**929.9**

How to choose the parameters in recommendation algorithms is an important issue in practice, especially when the algorithm has several parameters. If the optimal parameters vary significantly over time in real systems, the recommendation algorithm might not be meaningful from practical point of view. To test our algorithms in this aspect, we consider the triple division of the data. The data is randomly divided into three parts: the training set contains 80% of the links, another 10% forms the testing set and the remaining 10% of data constitutes the probe set. Both the training set and testing set are treated as known data (“historical data”) and the testing set is used to estimate the optimal parameters for the recommendation algorithm. We run the recommendation algorithm on the training set and choose the parameters when the recommendation accuracy (*RS*) in the testing set is optimized. The parameters will be considered as the optimal parameters to apply to the “future” (the probe set). We compare the H-Hybrid method (with two parameters: λ_1_ and λ_2_) to the O-Hybrid method (with one parameter λ) in this three-fold data division in Table 2. Obviously, even though our method has one more parameter, the recommendation performance in both accuracy and diversity is better than the O-Hybrid method.

**Table 2:** The results of all the metrics for the O-Hybrid and H-Hybrid algorithms under the three-fold data division. The entries corresponding to the best performance over all methods are emphasized in black.

Network	Method	*RS*	*P*(20)	*H*(20)	*I*(20)
Movielens	O-Hybrid	0.0766	0.1239	0.8688	210.8
	H-Hybrid	**0.0755**	**0.1262**	**0.8865**	**202.7**
Netflix	O-Hybrid	0.0463	0.1205	0.8305	1330
	H-Hybrid	**0.0412**	**0.1356**	**0.9107**	**1168**
RYM	O-Hybrid	0.0630	0.0642	0.9268	925.2
	H-Hybrid	**0.0618**	**0.0675**	**0.9431**	**834.9**

## Discussion

The amounts of data made available by modern World Wide Web sites far exceed the information capability of any individual. Based on the mass diffusion and heat conduction processes, many diffusion-based methods have been designed to generate both accurate and diverse recommendation for online users [5]. Such kind of methods are usually based on two steps of diffusion on user-object bipartite networks. To carry out the recommendation for a target user, the resource starts from each object selected by the target user and diffuses first to the neighboring users then from these users to their selected objects. The objects with the highest final resource will be recommended to the target user. Motivated by the observed significant difference in the topological properties between user nodes and object nodes, we propose in this paper a heterogeneous diffusion method in which each diffusion step is controlled by a separate parameter. We find that the new method can achieve better recommendation performance than the state-of-the-art methods.

The novelty of this work is threefold. Firstly, it highlights the asymmetric nature of the bipartite networks. In H-Hybrid method, optimal λ_2_ is smaller than optimal λ_1_. It indicates that the diffusion from users to items should be based more on the diversity-favoring diffusion process, while more weight should be put on the accuracy-favoring diffusion process when resource diffuses from items to users. Secondly, the accuracy of the diffusion-based recommendation algorithms is further improved. After many efficient methods were proposed, researchers realize it is now very difficult to further improve the accuracy of diffusion-based recommendation algorithms. The research focus recently has shifted to how to further enhance the recommendation diversity by designing new diffusion-based recommendation algorithms [34]. In this paper, we show that the recommendation accuracy can be further improved once the heterogeneous diffusion process is introduced. Finally, the heterogenous diffusion approach is not only restricted in the three methods in the paper (preferential diffusion, biased heat conduction, hybrid diffusion), it is actually very general and can be used to improve many other diffusion-based recommendation algorithms with parameters.

We remark that the idea of heterogeneous diffusion can be applied to many other problems. For example, in the well-known HITS ranking algorithm [35], each node’s authority score is equal to the sum of the hub scores of each node that points to it, and each node’s hub score is equal to the sum of the authority scores of each node that it points to. One can modify the above two iteration steps to obtain a more objective ranking results. One possible way to realize this idea is to introduce different nonlinear forms when summing the scores from neighboring nodes in the HITS algorithm. Some works actually have already been done in this direction [36, 37]. Moveover, diffusion processes have been widely used to solve many problems in complex networks such as link prediction [38], community detection [39] problems. The heterogeneous diffusion process may further improve the performance of these methods.

## Supporting Information

S1 File The degree correlation in the original network and the reshuffled networks (Figure A), the results of all the metrics for different recommendation algorithms (Table A). (PDF)

S2 File The data of real networks used in this paper. (RAR)
