# Supplementary material for: Information Filtering via Heterogeneous Diffusion in Online Bipartite Networks
Source: PLoS One. 2015 Jun 30;10(6):e0129459. doi: 10.1371/journal.pone.0129459 (PMC4488376; doi:10.1371/journal.pone.0129459)
Supplement: S1 File — (PDF) [file pone.0129459.s001.pdf]

Supplementary Information

Information filtering via heterogeneous diffusion in online bipartite networks

Fu-Guo Zhang and An Zeng

Degree correlation in bipartite networks.

In the main paper, we denote k_i as the degree of user i and d_i as the average degree of the objects selected by user i. All the users with the same degree k are selected and their d values are averaged to obtain $d(k)$. We find that $d(k)$ is decreasing with k (see fig. 1 in the main paper). In order to understand better this phenomenon, we compare the results of the real network with the reshuffled networks. The reshuffling process is based on many steps of link swapping. In each step, two links A-B and C-D are randomly selected and changed to A-D and C-B. In this way, the degree of nodes is preserved and the network is randomized. The comparison is shown in Figure A. One can see that the negative correlation in the reshuffled network still exists. This indicates that the negative correlation is partially determined by the degree of nodes. More specifically, popular objects are popular among many users (both those of with a high degree and those with a low degree). After exhausting the selection of popular objects, users that select more objects select from the remaining less popular objects.

However, we also notice that the curve of the reshuffled networks is flatter than that of the original network. This is more obvious in sparser network (the RYM network). This result suggests that the negative correlation observed in real network is also due to the fact that "high degree users tend to select unpopular objects while small degree users trend to select popular objects."

Comparison to the Directed Weighted Conduction method.

The methods we compared in the paper are quite recent. The O-Hybrid method was proposed in 2010, and the PD and BHC methods are proposed in 2011. In more recent years, new diffusion-based methods are mainly designed to improve the recommendation diversity. In order to compare with the newest methods, we consider a representative diffusion-based method proposed in 2014. The comparison of this method (denoted as Directed Weighted Conduction, short for DWC) to our methods is shown in table A. One can see that DWC can indeed outperform the heterogeneous diffusion (H-Hybrid, H-PD, H-BHC) in diversity, but some amount of recommendation accuracy is sacrificed.

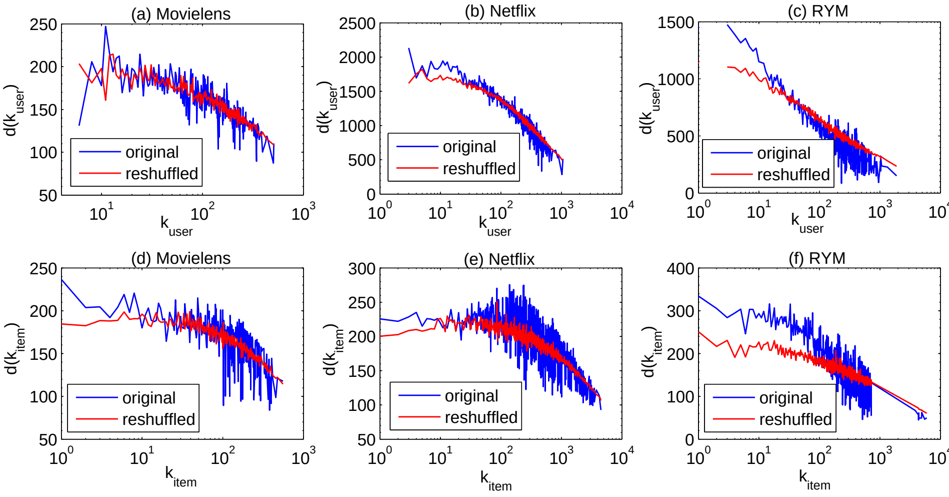

Figure A: (Color online) the degree correlation in the original network and the reshuffled networks.

Table A: The results of all the metrics for different recommendation algorithms. The entries corresponding to the best performance over all methods are emphasized in black.

Network	Method	RS	$P(20)$	$H(20)$	$I(20)$
Movielens	O-Hybrid	0.0733	0.1545	0.8735	230.9
	H-Hybrid	0.0717	0.1573	0.8919	221.4
	PD	0.0703	0.1602	0.8831	225.8
	H-PD	**0.0701**	**0.1621**	0.8905	222.5
	BHC	0.0753	0.1510	0.8603	238.4
	H-BHC	0.0741	0.1544	0.8833	230.3
	DWC	0.0826	0.1035	**0.9368**	**109.6**
Netflix	O-Hybrid	0.0447	0.1561	0.8404	1466
	H-Hybrid	**0.0395**	**0.1775**	0.9160	1285
	O-PD	0.0406	0.1485	0.8486	1324
	H-PD	0.0405	0.1461	0.9088	1024
	O-BHC	0.0474	0.1522	0.8550	1365
	H-BHC	0.0448	0.1708	**0.9402**	1085
	DWC	0.0443	0.0908	0.9267	**622**
RYM	O-Hybrid	0.0606	0.0727	0.9239	1060
	H-Hybrid	0.0586	0.0750	0.9326	1012
	O-PD	0.0588	0.0755	0.9359	987.3
	H-PD	**0.0588**	**0.0755**	0.9359	987.3
	O-BHC	0.0651	0.0645	0.9281	966.7
	H-BHC	0.0646	0.0665	0.9342	929.9
	DWC	0.0619	0.0705	**0.9657**	**659.6**
